# Biochemical profiling of the follicular environment to predict oocyte competence in cattle

**DOI:** 10.1371/journal.pone.0298316

**Published:** 2024-03-11

**Authors:** Nayara Ribeiro Kussano, Mauricio Machaim Franco, Margot Alves Nunes Dode

**Affiliations:** 1 Institute of Biology, University of Brasilia, Brasília-DF, Brazil; 2 Laboratory of Animal Reproduction, Embrapa Genetic Resources and Biotechnology, Brasília-DF, Brazil; 3 School of Veterinary Medicine, Federal University of Uberlândia, Uberlândia, Minas Gerais, Brazil; 4 Institute of Biotechnology, Federal University of Uberlândia, Uberlândia, Minas Gerais, Brazil; China Agricultural University, CHINA

## Abstract

To identify markers of oocyte competence, we compared the biochemical characteristics of fluid and cells from follicles containing oocytes with different capacities to form an embryo. Follicles (5–6 mm) were dissected, and follicular fluid (FF), granulosa cells (GC), cumulus cells (CC) from immature and mature cumulus-oocyte-complexes (COC) were individually collected. The oocytes were matured, fertilized, and cultured individually until day 8 (D8) of development. On D8, the samples were grouped according to embryo production into those that gave rise to blastocysts (EMB) and those that did not reach the blastocyst stage (NEMB). In CCs from immature and mature COCs and GCs, expression of CASP3, SERPINE2, VCAN, LUM, FSHR, EGFR, PGR, and GHR genes was quantified. Cell-free DNA (cfDNA), progesterone, and estradiol concentrations in the FF were determined. Data were analyzed by Mann–Whitney U test (GraphPad Prism 9). GHR was highly expressed in immature CCs from the EMB group, whereas CASP3 was highly expressed in mature CCs from the NEMB group (P<0.05). During maturation, the expression of CASP3 and GHR genes increased only in the NEMB group. ART2 cfDNA was highly detected in FF of the NEMB compared to the EMB group. Progesterone concentration was similar between the groups, whereas estradiol concentration was higher (P<0.05) in the EMB than in the NEMB group. It was concluded that a higher level of GHR transcripts in immature CCs, lower CASP3 expression in CCs from matured COCs, lower levels of ART2, and higher estradiol concentrations in FF may indicate oocytes with greater potential for development.

## Introduction

Oocyte competence is the main factor that determines the success of in vitro embryo production (IVEP) [1]. This competence is gradually acquired during oogenesis through a range of cellular and molecular attributes that provide the oocyte with the capacity to complete meiosis, fertilize, and develop into a viable embryo.

In domestic animals, immature oocytes used in IVEP are prematurely removed from a heterogeneous population of follicles that show different degrees of competence. Therefore, only a portion of the recovered oocytes can develop into embryos and have a normal pregnancy [2, 3]. An alternative to optimize the efficiency of IVEP is to select and use only the most competent oocytes. However, morphological assessment, which is a routinely used criterion for selecting immature oocytes for IVEP, cannot discriminate between more competent and less competent oocytes [3–5]. Thus, other selection criteria, such as molecular markers that more accurately indicate oocytes with the greatest potential to form an embryo, can be useful for improving IVEP.

A healthy follicular environment is essential for the oocyte to acquire competence and ensure successful fertilization and embryonic development [6, 7]. This environment depends on the balanced contribution of various components such as hormones, growth factors, electrolytes, metabolites, and proteins. Thus, follicular components, such as follicular fluid (FF) and follicular cells may reflect oocyte quality and represent potential sources of molecular markers for competence.

FF is a complex biological fluid comprising various components such as electrolytes, RNAs, proteins, amino acids, peptides, sugars, hormones, growth factors, metabolites, and extracellular vesicles. It is an autocrine and paracrine communication route between the theca, granulosa cells (GC), cumulus cells (CC), and the oocyte [8, 9]. Its composition is related to the stage of follicular growth and developmental potential of the oocyte [8, 10]. Some components such as Glycine, L-glutamate, L-alanine, glucose, pyruvate, estradiol, and progesterone have been identified as indicators of oocyte quality [11–13]. Recently, several studies have demonstrated the presence of cell-free DNA (cfDNA) and specific microRNAs (miRNAs) in FF [14–19]. In addition to FF, CC and follicular cells are also part of the follicular environment and communicate directly or indirectly with oocytes. Therefore, their physiological state may also reflect the status of the oocytes. Several studies have focused on identifying molecular markers of CC [20–24], and several differentially expressed genes have been identified in the CC of cumulus-oocyte complexes (COCs) with different levels of competence and are considered candidates for molecular markers of oocyte competence [11, 20, 22–25]. Although a few studies have focused on GC to identify markers of oocyte competence, these cells have also been reported to be a potential source of molecular markers [26, 27].

Furthermore, although several studies have identified parameters related to oocyte quality and possible markers, there is still no index, variable, or combination of variables that can be used to safely select the most competent oocytes. Knowledge of the differences between follicles that contain oocytes with greater or lesser developmental potential may help identify markers for competence and biochemical characteristics that could be mimicked *in vitro* to improve culture conditions. Therefore, the present study aimed to evaluate and compare a set of biochemical parameters of the follicular environment in follicles containing oocytes of different competencies.

## Experimental design

The aim of this study was to identify the biochemical characteristics of follicles containing oocyte with high developmental competence. Two groups were formed according to the IVP outcomes: a group in which oocytes gave rise to blastocysts (EMB) and a group that did not form blastocysts (NEMB). Three experiments were conducted using the same experimental groups. A total of 216 follicles were dissected, and all follicular components were collected and stored individually. Experiment 1 evaluated the number of transcripts of candidate genes in CC and GC from follicles containing oocytes with high and low capacities to produce embryos *in vitro*. In experiment 2, the amount of cell-free DNA (cfDNA) in FF from follicles containing oocytes with high and low capacity to produce embryos *in vitro* was quantified, and in experiment 3, the concentrations of progesterone and estradiol in FF samples were determined according to the IVP outcomes.

## Materials and methods

Unless otherwise indicated, all reagents and chemicals used in this study were purchased from Sigma-Aldrich (St. Louis, MO, USA).

All material used in the present study were aspirated from slaughterhouse ovaries and all the procedures were performed in accordance with the Brazilian Law for Animal Protection (*No*. *10*,*468 (2020)*. Ethical approvals are not required because only cumulus-oocyte-complexes, obtained from bovine ovaries collected at commercially slaughtered house were used.

### Collection and processing of samples

Ovaries were collected from local abattoirs immediately after slaughter and transported to the laboratory in saline solution (0.9% NaCl) supplemented with antibiotics (100 IU/mL penicillin G and 100 μg/mL of streptomycin) at 32–35°C. The time between ovary collection at the slaughterhouse and oocyte placement for maturation did not exceed 4 h.

Follicles were dissected using scalpels, tweezers, and scissors at room temperature [20]. During dissection, follicles were placed in washing medium (TCM-199 with Hank’s salts supplemented with 0.4% BSA) on a hot plate maintained at 36°C. Thus, they were measured using a graduated eyepiece (OSM-4, Olympus, Tokyo, Japan), and only cumulus-oocyte complexes (COCs) from 5–6 mm diameter-sized follicles, with a homogenous cytoplasm, and at least four layers of CCs were used.

#### Collection of follicular fluid and granulosa cells

The dissected and selected follicles were individually transferred to a petri dish, where they were ruptured individually with the aid of a needle (26G). After rupture, FF and GC were transferred to a 0.2 mL microtube. Tubes containing the individual samples were centrifuged at 300 × *g* for 10 min at 4°C to separate FF and cell fractions. The supernatant (FF) was removed and transferred to another microtube, and a 5 μL sample of FF was stored individually at −80°C for hormonal analysis; the remainder was centrifuged again at 2000 × *g* for 10 min and stored at −80°C for analysis of gene expression. After centrifugation, supernatant was transferred to another microtube and centrifuged once more at 16500 × *g* for 30 min at 4°C. The supernatant was then individually stored at −80°C. The pellet resulting from the first centrifugation, containing the GC, was washed twice in phosphate buffer (PBS), centrifuged at 3000 × *g* for 2 min, and stored in RNAlater (Ambion® Life Technologies, Carlsbad, CA, USA) at −80°C.

#### Biopsies of cumulus cells

After being released from the follicle, immature COCs were individually transfer to 50 μL drops of FF (previously centrifuged at 700 × *g* for 5 min). A small fragment of CCs was removed using a needle (26G), and COCs were immediately placed in maturation medium and incubated. After *in vitro* maturation, a new biopsy was performed on each COC to remove a small fragment of mature CCs using a needle (26G). The individual immature and mature biopsy samples were washed twice with 50 μL of PBS, identified, and stored in RNAlater (Ambion^®^ Life Technologies, Carlsbad, CA, USA) at −80°C until further use.

#### *In vitro* maturation, fertilization, and embryo culture

COCs were individually washed, transferred to the maturation medium, and cultured for 22 h at 38.5°C in 5% CO_2_ in air_._ Dishes with a diameter of 60 mm were prepared with 16 microdrops of 20 μL and covered with mineral oil. The maturation medium consisted of TCM-199 supplemented with 0.4% BSA–fatty acid-free, 0.01 IU/mL follicle-stimulating hormone (FSH), 0.1 mg/mL L-glutamine, 0.075 mg/mL amikacin, and 0.1 μM cysteamine.

Frozen semen from a Nelore bull with proven fertility was used for all treatments and replicates. Motile spermatozoa were obtained using a Percoll gradient (GE^®^ Healthcare, Piscataway, NJ, USA), as described by Machado et al. [28]. Following analysis of sperm concentration and motility, a solution of 350 μL of fertilization media with a final concertation of 1 × 10^6^ cells/mL was prepared. This solution was used to set up 16 drops (20 μL) in the culture plate to which the matured oocytes were individually transferred. Spermatozoa and oocytes were co-incubated for 18 h at 39°C in 5% CO_2_. Day 0 was considered as the day of *in vitro* insemination. The fertilization medium consisted of Tyrode’s albumin lactate pyruvate (TALP) [29] supplemented with 2 mM penicillamine, 1 mM hypotaurine, 250 mM epinephrine, and 10 μg/mL heparin.

After 18 h of co-incubation, the presumptive zygotes were washed and transferred to synthetic oviduct fluid (SOF) media with amino acids, citrate, and inositol (SOFaaci) [30]; supplemented 0.4% BSA; and incubated at 38.5°C in 5% CO_2_ for eight days. After washing, zygotes were transferred to a dish containing 16 microdrops (20 μL) of SOF medium maintaining the same positions they occupied on the fertilization dishes. Embryos were evaluated on day 2 for cleavage and on days 6, 7, and 8 to determine the blastocyst rates. On day 8 (D8), embryos were washed with PBS and stored individually in RNAlater at −80°C.

### RNA extraction, cDNA synthesis, and qPCR

Total RNA was isolated from four pools of each of the three cell types: immature CC, mature CC, and GC. Each pool contained ten samples collected from ten follicles.

The transcript levels of eight genes that have been investigated by our group for some years, and correlated in the literature with oocyte competence and quality: CASP3 (caspase 3) [31], SERPINE2 (serpina belonging to the E family, member 2) [32, 33], VCAN (versican) [11, 22, 34], LUM (lumican) [25], FSHR (follicle-stimulating hormone receptor) [20, 23], EGFR (epidermal growth factor receptor) [20, 32], PGR (progesterone receptor) [35], and GHR (growth hormone receptor) [20, 22, 23, 36], were quantified by qPCR. PCR was performed using the 7500 Fast Real-Time PCR System (Applied Biosystems, Foster City, CA, USA). Total RNA was isolated using the RNeasy Plus Micro Kit (Qiagen^®^, Hilden, Germany) according to the manufacturer’s instructions. Total RNA was used for cDNA synthesis using the GoScript Reverse Transcriptase Kit (Promega^®^, Madison, Wisconsin, USA) with Oligo-dT (0.5 μg/μL) and random primers (0.5 μg /μL) in a final volume of 30 μL. Reactions were performed at 70°C for 5 min, 25°C for 5 min, 42°C for 60 min, followed by enzyme inactivation at 70°C for 15 min. qPCR was performed using a GoTaq Master Mix Kit (Promega^®^). Reactions were optimized to provide the maximum amplification efficiency for each gene (Primer Efficiency 80–110%). Efficiencies were calculated using serial dilutions (1/4). Each sample was analyzed in triplicate, and the specificity of each PCR product was determined based on the melting curve analysis and amplicon size on an agarose gel. Reactions were performed in a final volume of 25 μL using cDNA equivalent to 0.35 of the CC biopsy and CG samples. PCR cycling conditions were 95°C for 5 min, followed by 50 cycles of denaturation at 95°C for 15 s and annealing and extension at 60°C for 30 s.

In a previous experiment, the amplification profiles of three constitutive genes, glyceraldehyde-3-phosphate dehydrogenase (GAPDH), β-actin (ACTB), and peptidylprolyl isomerase A (PPIA), were analyzed using GeNorm software [37], which indicated that GAPDH was the most stable gene. GAPDH was used as the reference for normalization. The relative expression of each gene was calculated using the ΔΔCt method with efficiency correction by the Pfaffl method [38]. The names, sequences, primer concentrations, and amplicon sizes for each gene are listed in Table 1.

**Table 1:** Primer sequences, primer concentration (nM), amplicon sizes in base pairs (bp), and reference GenBank accession numbers used for gene expression in qPCR.

Genes	Sequences	Primer concentration (nM)	Amplicon sizes (bp)	Reference GenBank
GAPDH	F 5` GGCGTGAACCACGAGAAGTATAA-3` R 5` CCCTCCACGATGCCAAAGT – 3`	300	119	NM_001034034.2
EGFR	F 5’ AAAGTTTGCCAAGGGACAAG 3’ R 5’ AAAGCACATTTCCTCGGATG 3’	300	253	XM_002696890.3
GHR	F 5’ AGAGATTCATGCCGACATCC 3’ R 5’ CGTTGTCTGGTTCTCACACG 3’	200	210	JQ711177.1
FSHR	F 5` GGATGCCATCATCGACTCTG 3` R 5` TGACTCGAAGCTTGGTGAGAAC 3’	300	133	NM_174061
PGR	F: 5’TCAGGCTGGCATGGTTCTTGG3’ R: 5’CTTAGGGCTTGGCTTTCGTTTGG3’	300	126	NM_001205356.1
LUM	F: 5’GTCTCCCAGTGTCTCTTCTAA 3’ R: 5’GAGATCCAGCTCCAACAAAG 3’	300	179	NM_173934.1
SERPINE2	F: 5’GACTCCTTTCCTACATCTTTCC 3’ R: 5’CAGTACAGTGTTCCACCATC 3’	300	158	NM_174669.2
CASP3	F: 5’GCCCAGGACTTTAGCAGTCA3’ R: 5’AAATGTGAGCGCCTTTGTT3’	300	185	NM_001077840.1
VCAN	F 5` TCATAGCCACCCCAGAGC 3` R 5` TTCCTTCCCCATCATGTCTC 3`	300	143	NM_181035

F = forward; R = reverse

### Extraction and quantification of cell-free DNA from follicular fluid

Because of the very small volume of FF samples, it was necessary to establish a DNA extraction protocol that would allow the use of reduced volumes to quantify cell-free DNA (cfDNA). This protocol was based on a salting-out procedure routinely used in our laboratory.

Before DNA extraction, 10 pg of a plasmid containing the green fluorescent protein (GFP) gene was added to each FF sample as an exogenous control. For cfDNA extraction, 30 individual samples of 15 μL of FF from each group were used. Samples were incubated at 95°C for 1 min then plunged to −196°C in liquid nitrogen. These steps were repeated 5 times. Next, 5 μL of 6 M NaCl was added to each sample, which were homogenized by successive pipetting, maintained on ice for 5 min, and then centrifuged at 13,000 rpm for 10 min at 4°C. The supernatant was then transferred to another microtube and 1/1 volume of ice-cold isopropanol was added. Following, samples were homogenized by pipetting and stored at −20°C for one hour. Samples were then centrifuged at 13,000 rpm for 15 min at 4°C, following which the supernatant was discarded, and the pellet was washed with the same volume of 70% ethanol and centrifuged again at 13,000 rpm for 10 min at 4°C. The resulting supernatant was discarded, and the pellet was dried in air for 10–15 min. Finally, the pellet was resuspended in 10 μL of DNase free water and stored at −20°C.

ART2 and Bov-tA short interspersed nuclear elements (SINEs), which comprise ~11.91% of the bovine genome [39] and are enriched in cfDNA [40], were used to quantify cfDNA in FF. The levels of ART2, Bov-tA, and GFP were evaluated by qPCR performed on a 7500 Fast Real-Time PCR System (Applied Biosystems, Foster City, California, USA). qPCR analysis was performed using the GoTaq Master Mix (Promega^®^). Each of the 30 FF samples was analyzed individually for both sequences, and the specificity of each PCR product was determined by melting curve analysis and amplicon size on an agarose gel. The reactions were performed in a final volume of 25 μL using cfDNA corresponding to 1/3 of each sample (3.3 μL) to amplify each gene (ART2, Bov-tA, and GFP). PCR cycling conditions were 95°C for 10 min, followed by 35 cycles of denaturation at 95°C for 15 sec and annealing and extension at 60°C for 1 min. To determine the levels of each target gene, the cycle threshold (Ct) value of each amplicon was divided by the Ct value of the reference gene (GFP), and this ratio was compared between the groups. The names, sequences, and concentrations of the primers and amplicon sizes of each gene are listed in Table 2.

**Table 2:** Primer sequences, concentration (nM), and amplicon sizes in base pairs (bp), used for cfDNA.

Genes	Sequences	Primer concentration (nM)	Amplicon sizes (bp)
ART2	F 5` CTCCCAGCATCAGAGTCTTT 3` R 5` CTGTGGTGTTGGAGAAGACT 3`	300	181
Bov-tA	F 5’ CTTCCCYTDGTRGCTCAG 3’ R 5’ CMAGRCTCCTCTGTCCATG 3’	150	148
GFP	F 5’ GGACAGCCTGAAGGAATTAG 3’ R 5’ CCAATCTGCTTAGTCACCTC 3’	300	100

F = forward; R = reverse

### Quantification of progesterone and estradiol in follicular fluid

Progesterone and estradiol concentrations were determined by chemiluminescence and electrochemiluminescence, respectively. Samples were analyzed in the commercial laboratory TECSA^®^ Tecnologia em Sanidade Animal, Belo Horizonte, MG, Brazil. Four pools, composed by 13 individual samples from each group totaling a volume of 100 μL each pool (50 μL for progesterone analysis and 50 μL for estradiol) were analyzed. The intra- and inter-assay coefficients of variation were 12.9% and 13.02% for progesterone, and 17.48% and 19.74% for estradiol, respectively. The sensitivity of the Estradiol assays was 0.009 to 3 ng/mL and for progesterone from 0.20 to 40 ng/mL, both at 1:10 dilution.

#### Statistical analysis

Quantification of gene expression that were normally distributed were analyzed using unpaired Student’s t-test, while those that were not normally distributed were evaluated using the Mann-Whitney test, for each group, four pools of ten samples (Immature/ mature cumulus cells and Granulosa cells) were used, evaluated in triplicate. Quantification of cfDNA and comparisons between hormone concentrations were performed using the Mann–Whitney test. All analyses were performed using GraphPad Prism 9 software (GraphPad Software, San Diego, California, USA).

## Results

**Fig 1:**
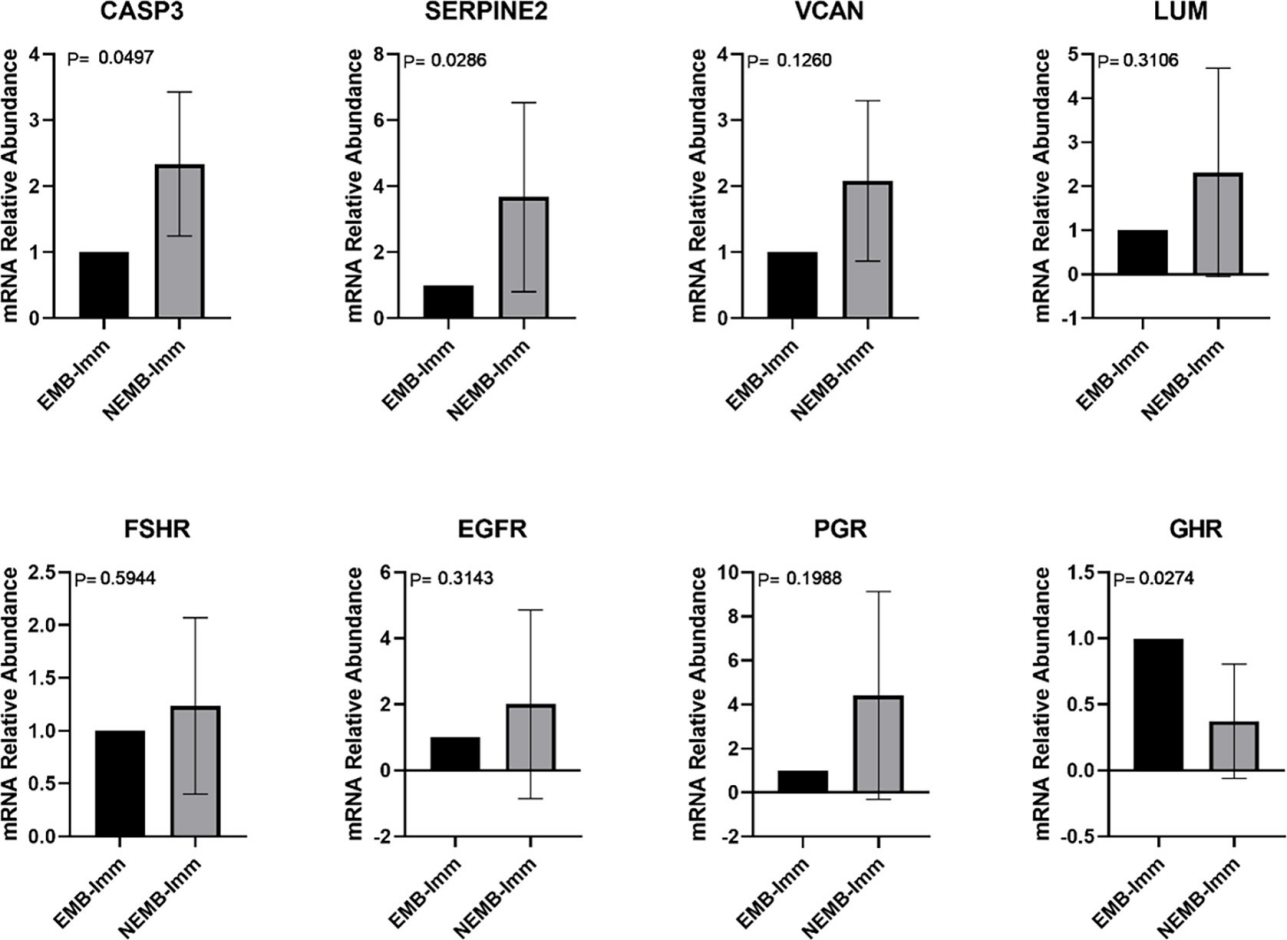
Transcripts levels of CASP3 (Caspase 3), SERPINE2 (Serpina belonging to the E family, member 2), VCAN (Versican), LUM (Lumican), FSHR (Follicle Stimulating Hormone Receptor), EGFR (Epidermal Growth Factor Receptor)), PGR (Progesterone Receptor), and GHR (Growth Hormone Receptor) genes in cumulus cells collected from cumulus-oocyte complexes (COCs) that gave rise to an embryo on D8 (EMB-Imm) and from COCs that cleaved but did not reach the blastocyst stage (NEMB-Imm). For each group, four pools of ten samples were used, evaluated in triplicate. Data are expressed as the mean ± standard deviation.

In the first experiment, the expression of genes related to oocyte competence in immature CC, mature CC, and GC from the two experimental groups, EMB and NEMB, was evaluated. Initially, the number of transcripts in the immature CC of the two groups was analyzed (Fig 1). CASP3 and SERPINE2 genes showed a greater expression in the NEMB group; in contrast, GHR showed a higher expression in the EMB group.

**Fig 2:**
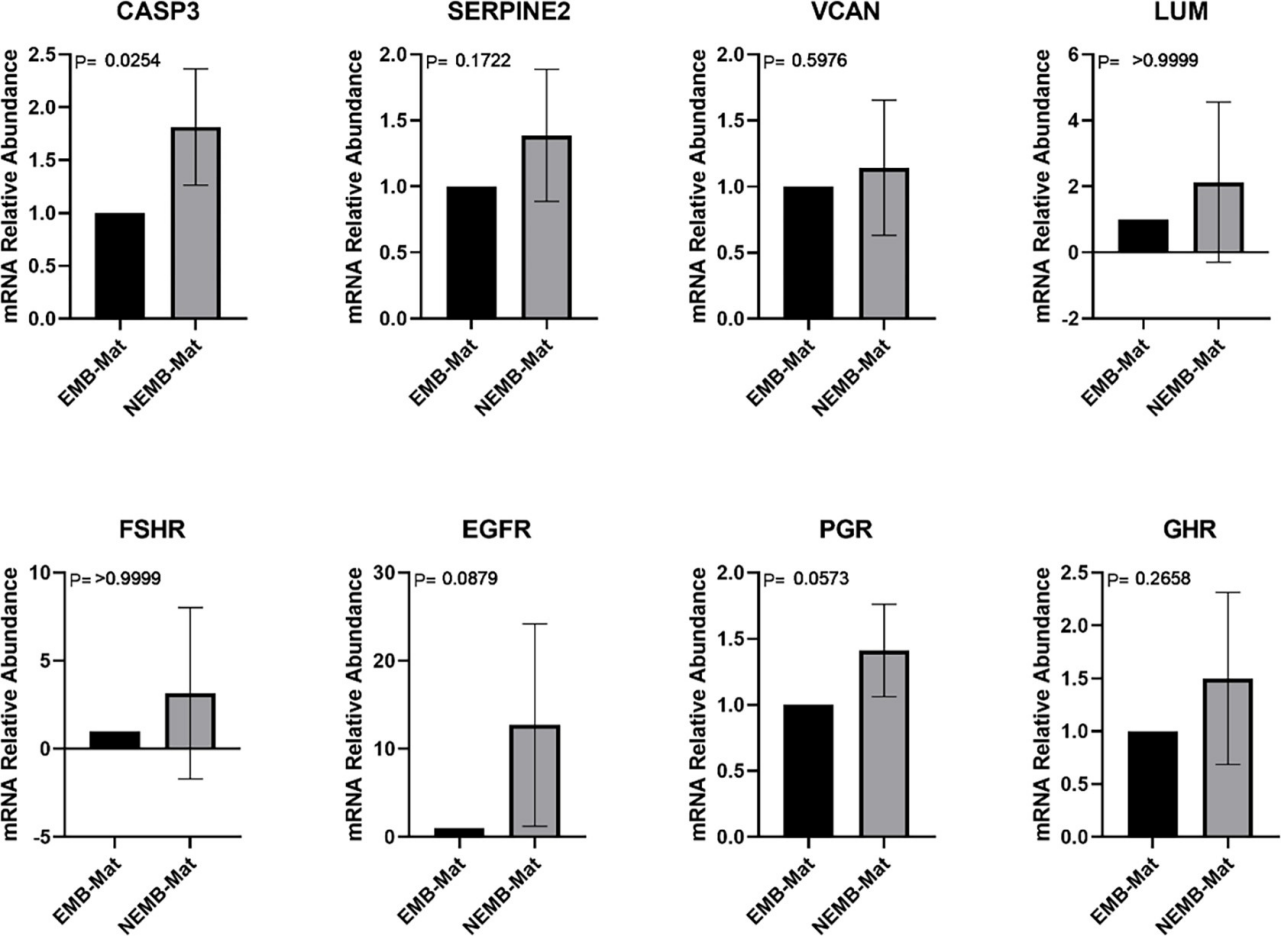
Transcripts levels of CASP3 (Caspase 3), SERPINE2 (Serpina belonging to the E family, member 2), VCAN (Versican), LUM (Lumican), FSHR (Follicle Stimulating Hormone Receptor), EGFR (Epidermal Growth Factor Receptor)), PGR (Progesterone Receptor), and GHR (Growth Hormone Receptor) genes in cumulus cells collected from mature cumulus-oocyte complexes (COCs) that gave rise to blastocysts on D8 (EMB-Mat) and from COCs that cleaved but did not reach the blastocyst stage (NEMB-Mat). For each group, four pools of ten samples were used, evaluated in triplicate. Data are expressed as the mean ± standard deviation.

Subsequently, the number of transcripts in CC after *in vitro* maturation of COCs was analyzed (Fig 2). The results showed that only CASP3 showed differential expression with the NEMB group showing the highest number of transcripts.

**Fig 3:**
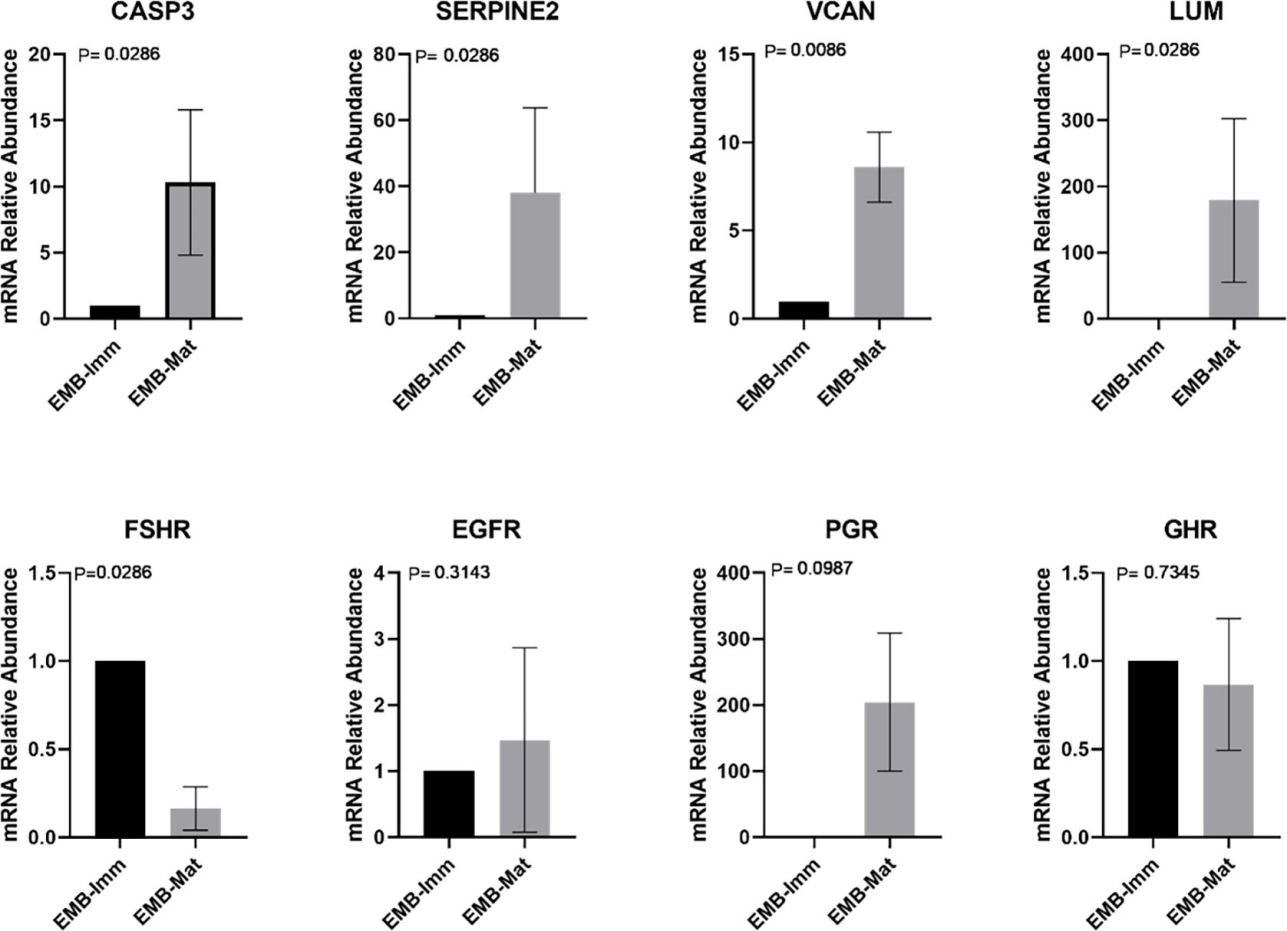
Transcripts levels of CASP3 (Caspase 3), SERPINE2 (Serpina belonging to the E family, member 2), VCAN (Versican), LUM (Lumican), FSHR (Follicle Stimulating Hormone Receptor), EGFR (Epidermal Growth Factor Receptor)), PGR (Progesterone Receptor), and GHR (Growth Hormone Receptor) in cumulus cells (CCs) collected from immature cumulus-oocyte complexes (COCs) that gave rise to an embryo on D8 (EMB-Imm) and in CCs collected from matured COCs that gave rise to an embryo on D8 (EMB-Mat). For each group, four pools of ten samples were used, evaluated in triplicate. Data are expressed as the mean ± standard deviation.

**Fig 4:**
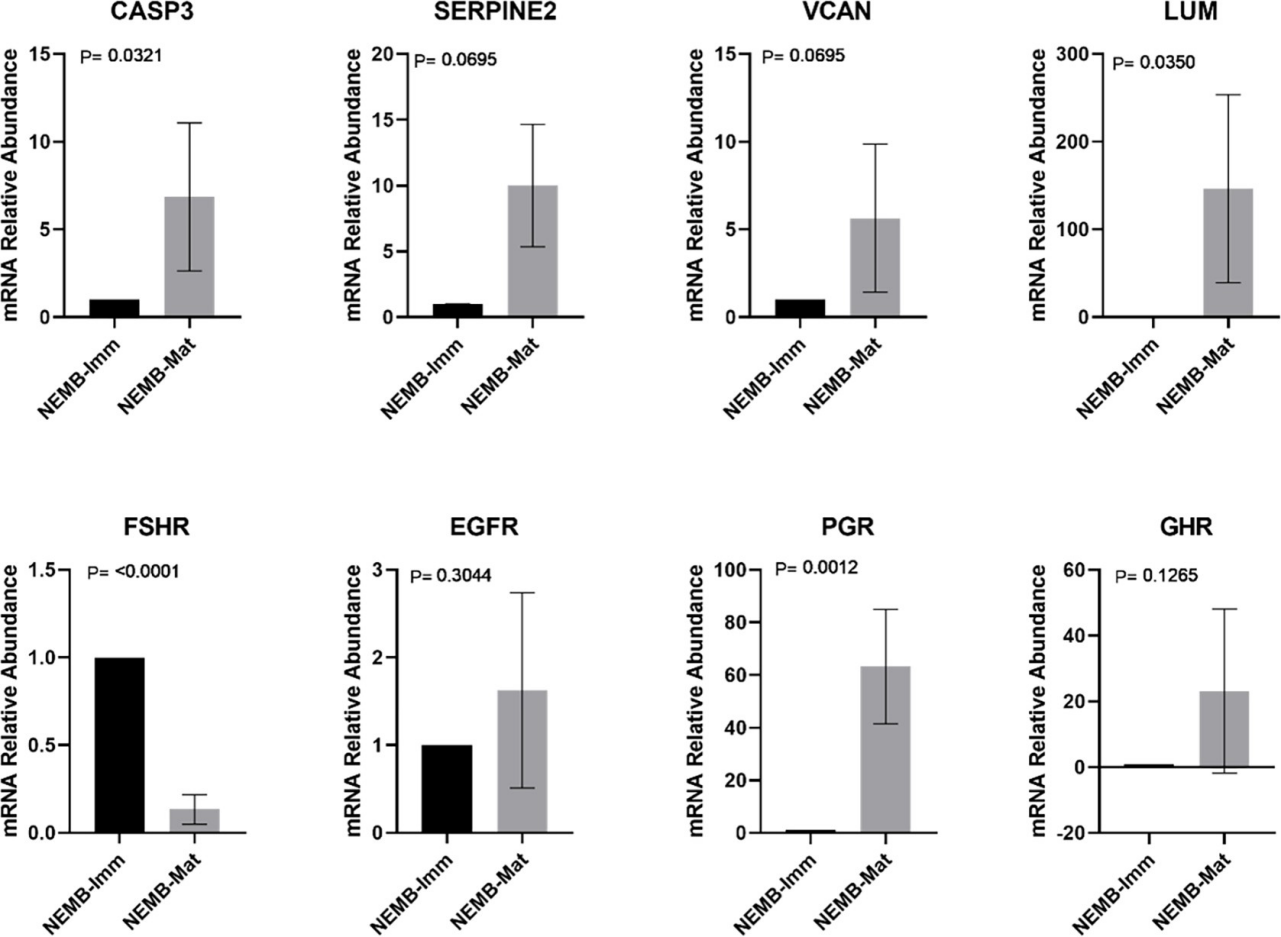
Transcripts levels of CASP3 (Caspase 3), SERPINE2 (Serpina belonging to the E family, member 2), VCAN (Versican), LUM (Lumican), FSHR (Follicle Stimulating Hormone Receptor), EGFR (Epidermal Growth Factor Receptor)), PGR (Progesterone Receptor), and GHR (Growth Hormone Receptor) in cumulus cells (CCs) from immature cumulus-oocyte complexes (COCs) that cleaved but did not reach the blastocyst stage (NEMB-Imm) and in CCs from mature COCs that cleaved but did not reach the blastocyst stage (NEMB-Mat). For each group, four pools of ten samples were used, evaluated in triplicate. Data are expressed as the mean ± standard deviation.

Finally, the transcript levels of all genes were evaluated during IVM (Figs 3 and 4). In the group in which COC originated in embryos (EMB-Mat), it was observed that in the majority of genes, except for PGR, GHR and EGFR, transcript levels changed during maturation. In the NEMB-Mat group, only the transcript levels of CASP3, LUM, PGR and FSHR changed during maturation.

**Fig 5:**
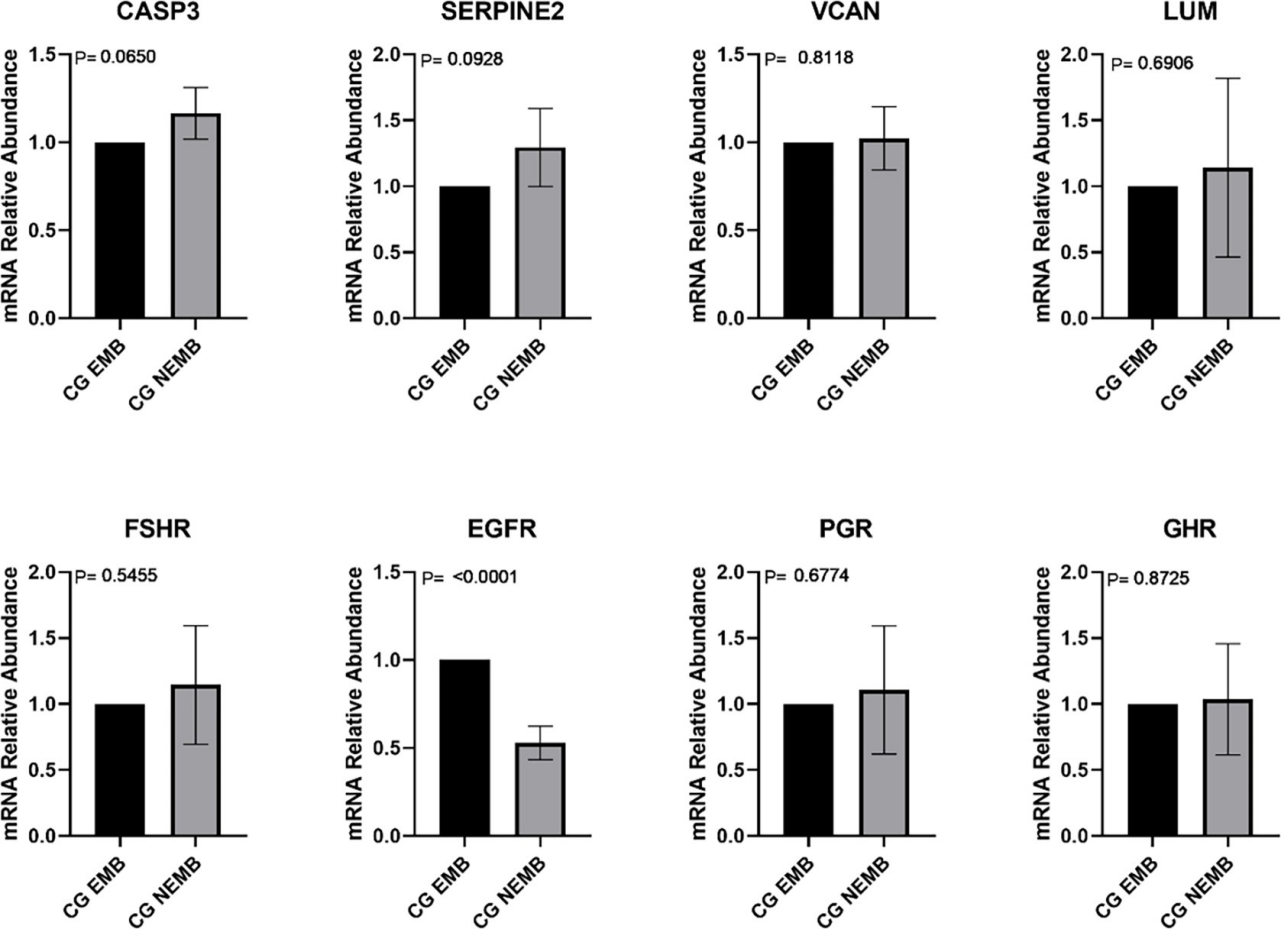
Transcripts levels of CASP3, SERPINE2, VCAN, LUM, FSHR, EGFR, PGR, and GHR in granulosa cells from follicles containing cumulus-oocyte complexes (COCs) that gave rise to an embryo on D8 (CG EMB) and from COCs that cleaved but did not reach the blastocyst stage (GC NEMB). For each group, four pools of ten samples were used, evaluated in triplicate. Data are expressed as the mean ± standard deviation.

Transcript levels of the selected genes were also compared in GC from follicles containing COCs from both experimental groups. Only EGFR showed significant differences between groups (Fig 5).

**Fig 6:**
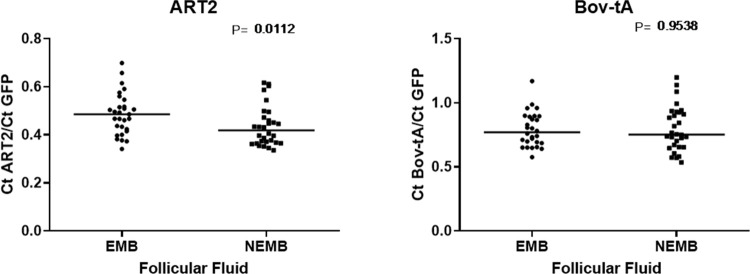
Quantification of ART2 and Bov-tA elements in follicular fluid samples collected from follicles containing cumulus-oocyte complexes (COCs) that developed to embryo on D8 (EMB) and COCs that cleaved but did not reach the blastocyst stage on D8 (NEMB). Each group consisted of 30 samples. Data are expressed as the mean ± standard deviation. Ct–Cycle threshold.

In the second experiment, cfDNA from the ART2 and Bov-tA elements was quantified. The results showed a greater amount (P<0.05) of cfDNA from ART2 in the FF of the NEMB group than that in the EMB group. For Bov-tA, no significant differences were observed (P>0.05) (Fig 6). To confirm the identify, ART2 and Bov-tA amplicons were sequenced, and the BLASTN and CLUSTAL tools were used. ART2 and Bov-tA amplicons showed 99% identity with Bos taurus genome (S1 and S2 Figs). These results confirm that ART2 and Bov-tA were present in follicular fluid samples.

**Fig 7:**
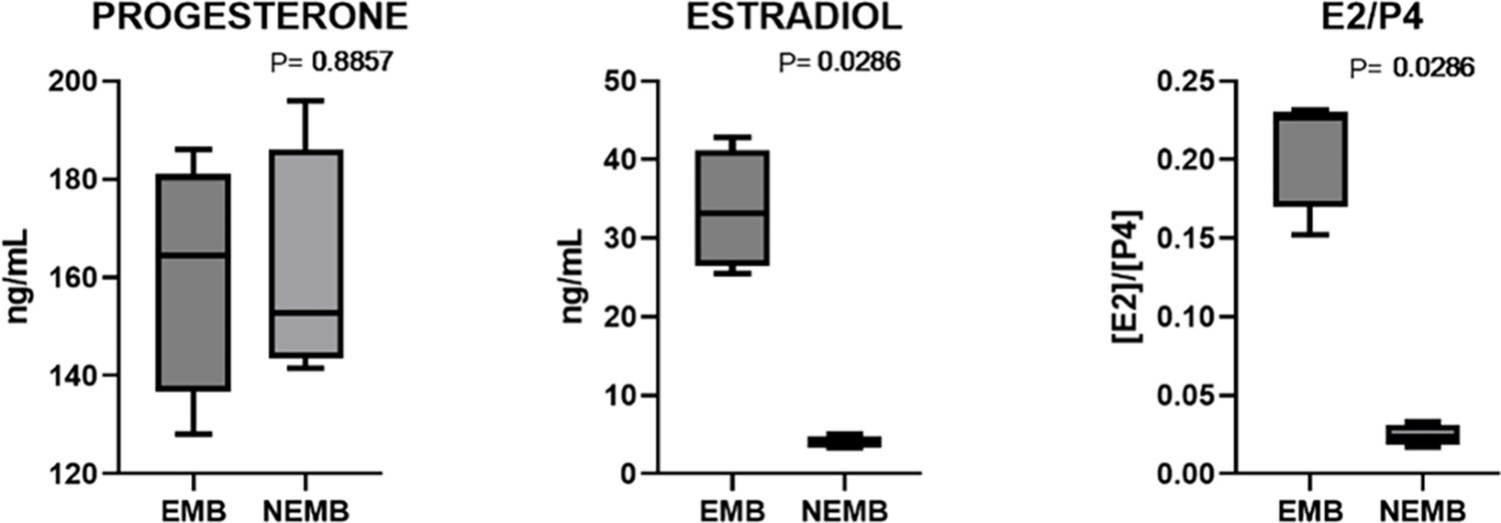
Concentration of progesterone and estradiol and their ratio (E2/P4) in follicular fluid from follicles that contained cumulus-oocyte complexes (COCs) that developed to embryo on D8 (EMB) and COCs that cleaved but did not reach the blastocyst stage on D8 (NEMB). For each group, four pools of 15 samples were analyzed. Data are expressed as the mean ± standard deviation.

In the third experiment, the concentrations of progesterone and estradiol in the FF were evaluated. The results showed that progesterone concentrations were similar between the EMB (164.5 ng/mL) and NEMB (152.8 ng/mL) groups. The estradiol concentration and E2/P4 ratio were higher (P<0.05) in the EMB group (33.14 ng/mL; 0.226) than in the NEMB group (3.73 ng/mL; 0.024) (Fig 7).

## Discussion

Considering that the follicular environment is essential for the development and acquisition of oocyte competence, the identification of biochemical parameters that differentiate follicles containing oocytes with greater competence can be useful for improving IVP results. In the present study, we quantified the transcript levels of candidate genes in follicular cells, cfDNA, and steroid hormone levels in the FF of individual follicles containing oocytes with different levels of competence. To categorize the level of competence of COCs, we used individual cultures and IVEP outcomes.

In Experiment 1, we quantified the transcript levels of candidate genes mentioned in the literature as possible molecular markers of oocyte competence. In CC from immature COCs, CASP3, SERPINE2, and GHR showed differences in transcript levels between the groups. The levels of CASP3 and SERPINE2 transcripts were lower, whereas those of GHR transcripts were higher in CC from COC that reached the blastocyst stage on D8. Considering that the selection of genes was based on the literature, it was expected that a greater number of genes would show differential expression, which may be due to differences in IVM systems, such as differences in medium composition, temperature, oxygen tension, and follicle size. This effect was corroborated by the results of Kussano et al. [36], who showed that the protein source used in IVM affected the transcript levels of certain genes.

Previous studies by our group have also found differences in the transcripts of this gene in CC from immature COCs [20, 23]. In these studies, the amount of GHR transcripts was higher in cumulus cells from follicles ≥8.1 mm, which contained more competent COCs. Similar results have also been reported in other studies that showed greater expression of this gene in CCs of immature COC that gave rise to embryos after IVF [22, 36]. Thus, the level of GHR transcripts was used as a molecular marker of competence. The presence of GH in IVM medium accelerates maturation, improves embryonic development, induces CC expansion, and increases IVP rates [41, 42]. This beneficial effect of GH was observed only in the presence of CCs, suggesting that its effect was due to the presence of GH receptors in these cells. This explains why higher GHR expression is related to oocyte competence.

Regarding CC after IVM, only CASP3 showed differences, with higher levels of transcripts in CC than those in the NEMB group. This result agrees with other reports showing that the expression of these genes is increased in the CC of women with fertility problems [31] and that CC apoptosis is related to embryonic development impairment [43, 44]. Caspase 3 is a pro-apoptotic factor expressed in follicular cells. Apoptosis occurs physiologically during follicular atresia, luteolysis, and follicular development [45]. Therefore, it is possible that the greater amount of CASP3 transcripts in the NEMB group, both in mature CC and in GCs, indicates that these follicles could be at the beginning of atresia, which had already compromising, in some way, oocyte quality.

Another piece of information that may be relevant in studies on oocyte competence and gene expression is the behavior of gene expression during IVM [46, 47]. Here, we noted that the transcript levels of most genes changed during IVM for both groups, which was expected considering the events that occurred during this period and the function of the genes evaluated. Interestingly, SERPINE2, VCAN, EGFR and GHR were the genes that did not showed increased expression during IVM in the NEMB group. However, this change were observed in the EMB group. This result may suggest that lower quality COCs do not have enough SERPINE2, VCAN, EGFR and GHR increase the number of transcripts to support IVM processes. Two studies involving evaluation of gene expression in CC during IVM of pigs COCs, observed that there was difference in expression between CC of prepubertal animals (less oocyte competence) and cycling females (greater oocyte competence), where CC of sows had better rates of transcription changes during IVM compared to prepubertal gilts [46, 47]. Results that are in line with the present study, where CC originating from COC that did not give rise to embryos, did not show a significant increase in the number of transcripts of the genes SERPINE2, VCAN, EGFR and GHR during IVM. These genes are of great importance for oocyte maturation and competence, cumulus cell expansion and oocyte development.

In experiment 2, the amount of cfDNA present in FF was evaluated. Because of the minimum volume of FF required to extract cfDNA using a commercial kit, it was necessary to establish a homemade protocol. We established a protocol based on a salting-out procedure and demonstrated that very small volumes of FF can be used to access cfDNA. This protocol makes it possible to extract cfDNA from small volumes in a simple and economical manner. Due to the lack of studies quantifying cfDNA in cattle FF, the present study was based on reports in humans, which routinely use ALU sequences for cfDNA quantification [48–50]. However, ALU has not been found in ruminants, necessitating the search for other SINEs that could be used in cattle. Thus, we chose to use the ART2 and Bov-tA elements [39, 40]. We found a greater amount of ART2 cfDNA in the FF of the NEMB group than that of the EMB group. Similar results were reported in pigs, where high amounts of cfDNA in FF were related to poor-quality oocyte development, cell death, and GCs apoptosis [16]. Other studies in humans have also confirmed these findings, showing low amounts of cfDNA in the FF of women with high embryonic development [18, 49, 51–54]. It is important to emphasize that both pig and human studies were performed using different follicle sizes and oocyte development stages compared to our study, which suggests that cfDNA may be a more reliable parameter because it presents the same behavior at different stages of development [49, 54].

Finally, progesterone and estradiol concentrations were evaluated in the third experiment. A higher concentration of estradiol and a higher E2/P4 ratio were found in the EMB group than in the NEMB group. Studies have shown higher estradiol concentrations in the FF of women with positive pregnancy results [12, 55, 56]. Using bovine FF from 8 mm follicles, Matoba et al. [11] found no differences in the levels of P4 and E2 between groups that did or did not originate from an embryo. It is important to note that follicles with a diameter of 5–6 mm were used in our study. This stage is close to the moment when the dominant follicle is selected; therefore, it is possible that follicles with the highest E2 in the FF may be the future dominant follicles that would contain better oocytes. Alves et al. [13] found a higher concentration of E2 in FF from COCs originating from embryos with fast cleavage than in blastocysts with slow cleavage. In fact, it has already been shown that in the follicular selection phase, the GC of the future dominant follicle have a greater capacity to secrete estradiol than subordinate follicles [57].

Another important point that can be raised about the effect of E2 on oocyte competence is the important relationship between E2 and the expression of Natriuretic Peptide Receptor 2 (NPR2), which is the type C natriuretic peptide receptor (NPPC), which are essential to maintain meiotic arrest in oocytes, a mechanism that is essential during the acquisition of competence. Studies have shown that the presence of E2 in the medium had a beneficial effect on oocyte competence, promoting and maintaining the expression of NPR2 in CC [58–60]. Therefore, in our study, a greater amount of E2 in the FF could be acting on the expression of NPR2 in CC, improving the competence and development of these oocytes, originating from follicles with a higher concentration of E2.

In summary, we assessed which parameters of the follicular environment better reflected the quality of oocytes to be used as markers for competence. Taken together, these results suggest that the best follicular condition is one with greater GHR expression in immature CC, lower levels of CASP3 transcripts in CC after IVM, lower amounts of cfDNA, and higher estradiol dosage in FF. Considering that estradiol can protect follicular cells from apoptosis induced by oxidative stress [61, 62], it is possible that a higher concentration of E2 protects the follicle, which would reduce the amount of cfDNA and the expression of CASP3 in GC and after IVM; consequently, the oocytes would have greater development potential. These results can add other characteristics to the selection of more competent oocytes, which can be used in a non-invasive manner, bringing benefits to assisted reproductive techniques, including IVP. Based on our results, it can be concluded that the differentially expressed genes, GHR in immature CCs, CASP3 in mature CCs, ART2 cfDNA, and estradiol concentration in the FF and its relationship with progesterone (E2/ P4), are parameters that can be used for the selection of competent oocytes in cattle.

## Supporting information

S1 Fig Alignment of ART2 sequences using the BLASTN tool (A) and CLUSTAL tool (B). X82879.1 Artificial sequences DNA for ART 2 consensus.
(PDF)

S2 Fig Alignment of Bov-tA sequences using the BLASTN tool (A) and CLUSTAL tool (B). M11267.1 Bovine steroid 21-hydroxylase gene (P-450-c21) gene, complete cds; Z69241.2 Bos taurus partial cyp19 gene exon 1; XM_027516684.1 PREDICTED: Bos indicus x Bos taurus adenylate kinase 2 (AK2), transcript variant X1, mRNA; U63109.1 Bos taurus alpha-lactalbumin A precursor, gene, partial cds; X05380.1 Bovine thyroglobulin gene exon 1 and flanks.
(PDF)
