# Supplementary material for: Biochemical profiling of the follicular environment to predict oocyte competence in cattle
Source: PLoS One. 2024 Mar 11;19(3):e0298316. doi: 10.1371/journal.pone.0298316 (PMC10927144; doi:10.1371/journal.pone.0298316)
Supplement: S1 Fig — Alignment of ART2 sequences using the BLASTN tool (A) and CLUSTAL tool (B). X82879.1 Artificial sequences DNA for ART 2 consensus. (PDF) [file pone.0298316.s001.pdf]

Supporting Information

Biochemical profiling of the follicular environment to predict oocyte competence in cattle

Nayara Ribeiro Kussano[1]; Mauricio Machaim Franco[2, 3, 4]; Margot Alves Nunes Dode[1, 2*]

[1]Institute of Biology, University of Brasilia, Brasília-DF, Brazil

[2]Laboratory of Animal Reproduction, Embrapa Genetic Resources and Biotechnology, Brasília-DF, Brazil

[3]School of Veterinary Medicine, Federal University of Uberlândia, Uberlândia, Minas Gerais, Brazil

[4]Institute of Biotechnology, Federal University of Uberlândia, Uberlândia, Minas Gerais, Brazil

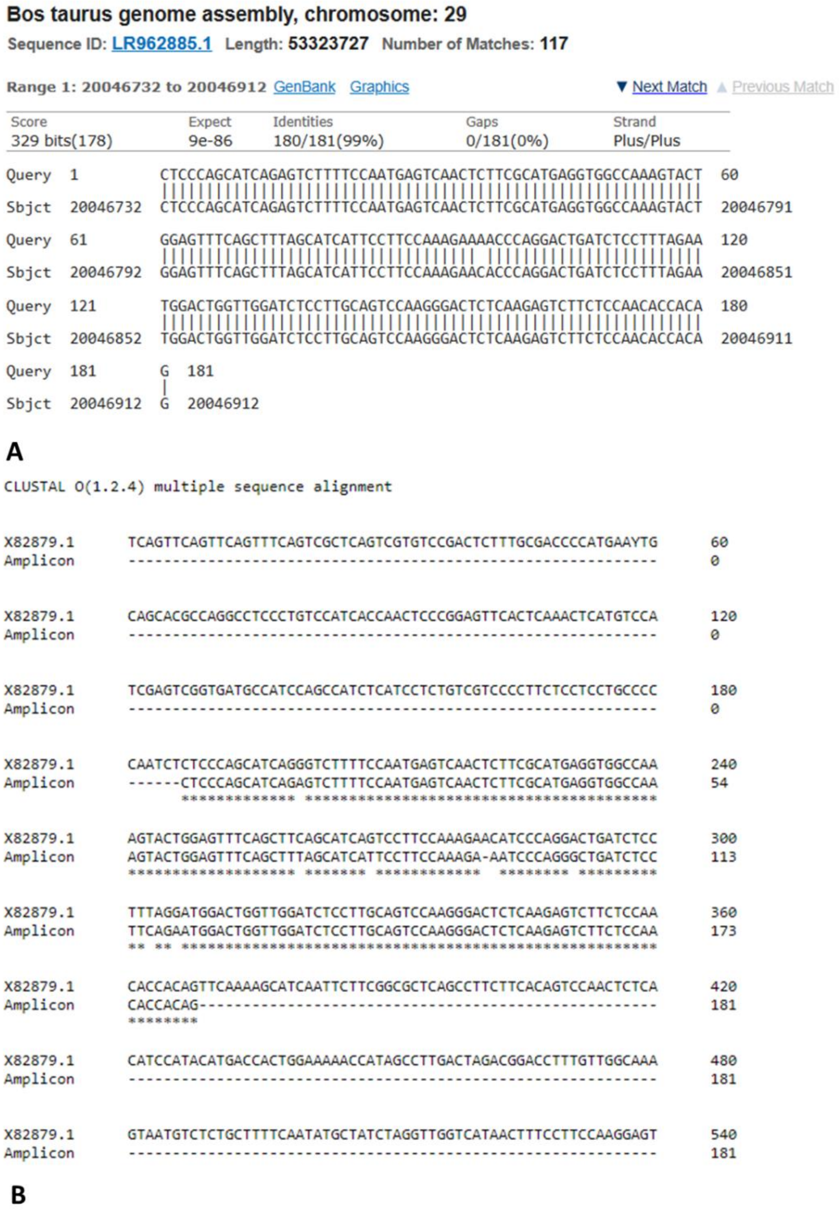

S1 Fig: Alignment of ART2 sequences using the BLASTN tool (A) and CLUSTAL tool (B).
X82879.1 Artificial sequences DNA for ART 2 consensus.
