# Supplementary material for: Biochemical profiling of the follicular environment to predict oocyte competence in cattle
Source: PLoS One. 2024 Mar 11;19(3):e0298316. doi: 10.1371/journal.pone.0298316 (PMC10927144; doi:10.1371/journal.pone.0298316)
Supplement: S2 Fig — Alignment of Bov-tA sequences using the BLASTN tool (A) and CLUSTAL tool (B). M11267.1 Bovine steroid 21-hydroxylase gene (P-450-c21) gene, complete cds; Z69241.2 Bos taurus partial cyp19 gene exon 1; XM_027516684.1 PREDICTED: Bos indicus x Bos taurus adenylate kinase 2 (AK2), transcript variant X1, mRNA; U63109.1 Bos taurus alpha-lactalbumin A precursor, gene, partial cds; X05380.1 Bovine thyroglobulin gene exon 1 and flanks. (PDF) [file pone.0298316.s002.pdf]

Supporting Information

Biochemical profiling of the follicular environment to predict oocyte competence in cattle

Nayara Ribeiro Kussano[1]; Mauricio Machaim Franco[2, 3, 4]; Margot Alves Nunes Dode[1, 2*]

[1]Institute of Biology, University of Brasilia, Brasília-DF, Brazil

[2]Laboratory of Animal Reproduction, Embrapa Genetic Resources and Biotechnology, Brasília-DF, Brazil

[3]School of Veterinary Medicine, Federal University of Uberlândia, Uberlândia, Minas Gerais, Brazil

[4]Institute of Biotechnology, Federal University of Uberlândia, Uberlândia, Minas Gerais, Brazil

Bos taurus genome assembly, chromosome: 22

Sequence ID: LR962878.1 Length: **61211244** Number of Matches: **93**

Range 1: 27612043 to 27612187 GenBank Graphics ▼ Next Match ▲ Previous Match

Score	Expect	Identities	Gaps	Strand
263 bits(142)	5e-66	144/145(99%)	0/145(0%)	Plus/Plus

```
Query  1         CTTCCCTGGTGGCTCAGACAGTAAAGAATCTGCCTGCAATGCAGGAGACCTGGGTTCTGT  60
                 ||||||||||||||||||||||||||||||||||||||||||||||||||||||||||||
Sbjct  27612043  CTTCCCTGGTGGCTCAGACAGTAAAGAATCTGCCTGCAATGCAGGAGACCTGGGTTCTGT  27612102

Query  61        CCCTGGGTCAGGAAAATCCCCTGGAGAAGGGAATGGCTCTCACTCCAGTATTCTTGCCTG  120
                 ||||||||||||||||||||||||||||||||||||||||||||||||||||||||||||
Sbjct  27612103  CCCTGGGTCAGGAAAATCCCCTGGAGAAGGGAATGGCTCTCACTCCAGTATTCTTGCCTG  27612162

Query  121       GAAAATTCCATGGACAGAGGAGCCT  145
                 ||||||||||||| |||||||||||
Sbjct  27612163  GAAAATTCCATGGATAGAGGAGCCT  27612187
```

A

```
CLUSTAL O(1.2.4) multiple sequence alignment


M11267.1          TGGTGGAAGTTTGGGCTTCCCTAGTGGCTCAGATGGTAAAGCATCTGCCTGTAATGCAGG       60
Z69241.2          TTAACAGTAATAGGGCTTCCCTTGTAGCTCAGTCAGTAAAGAATCTGCCTGCAGTGCAGG       60
XM_027516684.1    ---CTTTATTTTCGACTTCCCTGGTGGCTCAGACGGTAAAGCATCTGTCTACAATGCAGG       57
U63109.1          -------TTGTGGGGCTTCCCTGGTGGCTCAGATGGTAAAGTGTCTGCCTGCAATGTGGG       53
X05380.1          ------CCCCCAGTTCTTCCTTGGTGGCTCAGATGGTCAAGAATCCACCTGCAATGCGGG       54
Amplicon          ---------------CTTCCCTGGTGGCTCAGACAGTAAAGAATCTGCCTGCAATGCAGG       45
                                 ***** * ** ******    ** ***  **    **  * **  **

M11267.1          AGACCTAGGTTGGACCCCTGGCTGGGAAAGATGCCCTGAAGAAGAGAATGGCAACCCATT       120
Z69241.2          ATACCTGGGTTCAATCCCTGGGTTGGGAAGATCCCCTGGAGCAGGAAATGGCAACCCACT       120
XM_027516684.1    AGACCCGGGTTCGATCCCTGGGTTGGGAATATCCCCTGGAGAAGGAAATGGCAATCCACT       117
U63109.1          TGATCTGGGTTCGATCCCTGGCTTGGGAAGATCCCCTGGAGAAGGAAATGGCAACCCACT       113
X05380.1          AGACCTGGGTTTGATCCCTGGGTTGGGAAGATCCCCTGGAGAAGGGAATGGCTACCCACT       114
Amplicon          AGACCTGGGTTCTGTCCCTGGGTCAGGAAAATCCCCTGGAGAAGGGAATGGCTCT-CACT       104
                    * *  ****    ****** *  * ** ** ***** ** **  ******    ** *

M11267.1          CTAGTACTCTTGCCTGGCGAATTCCATGGACAGAGGAGTCTGGAGGGTTACAGTCCATGA       180
Z69241.2          CCAG------TGCCTGGAAAATCTCATGGACAGAGGAGCCTGGTGGGCTGCAGCCCATGG       174
XM_027516684.1    CTAGGACTGTTGCCTGGAAAATCCCATGGACAGAGGAGCCTGGTAGGCTACAGTCCATGG       177
U63109.1          CTAGTACTCTTACCTGGAAAATTCCATGGACAGAGGAGCCTTGTAAGCTACAGTCCATGG       173
X05380.1          CCAGTATTCTGGCCTGGAGAATCCCATGGACAGAGGAGCCTGGCGGGATGCAGTCCATGG       174
Amplicon          CCAGTATTCTTGCCTGGAAAATTCCATGGACAGAGGAGCCTTG----------------       147
                  * **        *****  ***  ************** ** *

M11267.1          GTTTGCAAAGAGTGGGACATGACTGAGTGACTGACTCACACACACACACACACACACACA       240
Z69241.2          GGTCGCAAAGAGTCGGGCACGACTGAGCGACTAACGCTAACACTTAACGGTAATATGA--       232
XM_027516684.1    GGTTGCAAAGAGTCAGACACAACTGAGCGACTTCACTTCACTTCACTTTATTTTCAA---       234
U63109.1          GATTGCAAAGAGTTGAACACAACTGAGCAACTAAGCACAGCACAGTACAGTATACACCTG       233
X05380.1          GGTCTCAGAGAGTCAGATGTGACTGAGCGACTTTCACACACATTCGTCCCTGGTT-----       229
Amplicon          ------------------------------------------------------------       147
```

B

S2 Fig: Alignment of Bov-tA sequences using the BLASTN tool (A) and CLUSTAL tool (B). M11267.1 Bovine steroid 21-hydroxylase gene (P-450-c21) gene, complete cds; Z69241.2 Bos taurus partial cyp19 gene exon 1; XM_027516684.1 PREDICTED: Bos indicus x Bos taurus adenylate kinase 2 (AK2), transcript variant X1, mRNA; U63109.1 Bos taurus alpha-lactalbumin A precursor, gene, partial cds; X05380.1 Bovine thyroglobulin gene exon 1 and flanks.
